# Bridging adjuvant treatment gaps: low and intermediate risk endometrial carcinoma patients care in Salah Azaiez Institute in Tunisia

**DOI:** 10.1093/oncolo/oyaf273

**Published:** 2025-09-17

**Authors:** Emna Boudhina, Semia Zarraa, Ghada Sahraoui, Alia Mousli, Khadija Ben Zid, Asma Ghorbel, Ameni Yousfi, Rim Abidi, Safia Yahiaoui, Chiraz Nasr

**Affiliations:** Radiation Therapy Department, Salah Azaiez Institute, Tunis, 1006, Tunisia; Radiation Therapy Department, Salah Azaiez Institute, Tunis, 1006, Tunisia; Anatomical Pathology Department, Salah Azaiez Institute, Tunis, 1006, Tunisia; Radiation Therapy Department, Salah Azaiez Institute, Tunis, 1006, Tunisia; Radiation Therapy Department, Salah Azaiez Institute, Tunis, 1006, Tunisia; Radiation Therapy Department, Salah Azaiez Institute, Tunis, 1006, Tunisia; Radiation Therapy Department, Salah Azaiez Institute, Tunis, 1006, Tunisia; Radiation Therapy Department, Salah Azaiez Institute, Tunis, 1006, Tunisia; Radiation Therapy Department, Salah Azaiez Institute, Tunis, 1006, Tunisia; Radiation Therapy Department, Salah Azaiez Institute, Tunis, 1006, Tunisia

**Keywords:** endometrial carcinoma, low risk, intermediate risk, guidelines adherence, adjuvant treatment, molecular era, low-resource settings

## Abstract

**Background:**

The management of low- and intermediate-risk endometrial carcinoma (EC) at the Salah Azaiez Institute (SAI) has evolved with international recommendations and the advent of the molecular era. We lack access to molecular profiling; consequently, both undertreatment and overtreatment are observed. This study explores how adherence to international recommendations affects clinical outcomes.

**Methods:**

We performed a retrospective analytic study including 180 women with stage I–II low-, low-intermediate-, and high-intermediate-risk EC treated at SAI between 2015 and 2020, with at least 3 years of follow-up. Ethical approval and patient consent were obtained; for telephone interviews, verbal consent was secured. We selected 60 women per risk group from a larger pool to ensure comparability. Eleven histopathologic slides exhibiting lymphovascular space invasion (LVSI) were reviewed independently by 2 experienced pathologists to distinguish focal from extensive LVSI; no slides without LVSI were reexamined. Risk stratification followed the 2020 ESGO–ESTRO–ESP guidelines, and adjuvant treatments were assessed according to the 2021 ESGO–ESTRO recommendations. Pelvic lymphadenectomy was the standard nodal assessment; sentinel lymph node (SLN) mapping was not available during the study period.

**Results:**

Median age was 60 years (range 35–69). After histopathologic review, 51.1% of tumors were grade 2, 54.5% had under 50% myometrial invasion, 8.3% had extensive LVSI, and 3.9% had focal LVSI. Forty-seven per cent of patients received guideline-concordant therapy, 47% were over-treated, and 6% were under-treated. Over-treated women had a 7.9-fold higher risk of death and a 6.6-fold higher risk of recurrence than appropriately treated women. Under-treatment was not a significant prognostic factor. Overtreatment and guideline-concordant therapy were both associated with higher rates of gastrointestinal, genitourinary, and sexual toxicities.

**Conclusion:**

Limited access to molecular profiling constrains personalized care in Tunisia. Strict adherence to current guidelines is essential to avoid unnecessary toxicity, and the integration of molecular classification and SLN mapping should be prioritized.

Implications for practice The identification of worse survival outcomes among the over-treated group and the heightened toxicity experienced by conformally and over-treated patients emphasize the critical need for unwavering adherence to international treatment guidelines. These guidelines serve as a cornerstone for establishing evidence-based and effective treatment protocols. However, the evolving landscape of cancer management, marked by the integration of molecular profiling, emerges as a crucial avenue for further refinement. Molecular profiling, by providing insights into the genetic and molecular characteristics of tumors, offers the potential to tailor treatments with greater precision. This personalized approach can optimize therapeutic efficacy while minimizing unnecessary interventions and improving patient outcomes. As such, the strategic integration of molecular profiling into clinical decision-making processes is pivotal for advancing the field of oncology and ensuring optimal patient care. Moreover, standardizing the use of volumetric modulated arc therapy is proposed as a strategic measure to mitigate the installation of treatment-related toxicities. Additionally, advocating for global standardization of histological reports, with a special focus on lymphovascular space invasion, is recommended to ensure uniformity and comparability in reporting practices across diverse regions, fostering improved communication and facilitating comprehensive data interpretation within the realm of international research and clinical collaboration.

## Introduction

Endometrial carcinoma (EC) is the most common gynecological malignancy in industrialized countries. International guidelines for low- and intermediate-risk disease have shifted from routine adjuvant therapy toward de-escalation and incorporation of molecular classification. However, implementing these advances is challenging in low-resource settings, where access to molecular profiling is limited. In Tunisia, EC management still relies on histological parameters. We therefore examined how adherence to contemporary ESGO–ESTRO–ESP guidelines influences patient outcomes in a representative cohort treated at Salah Azaiez Institute (SAI).^1,^^2^^,^^3^

## Materials and methods

### Study design and population

**Figure 1.**
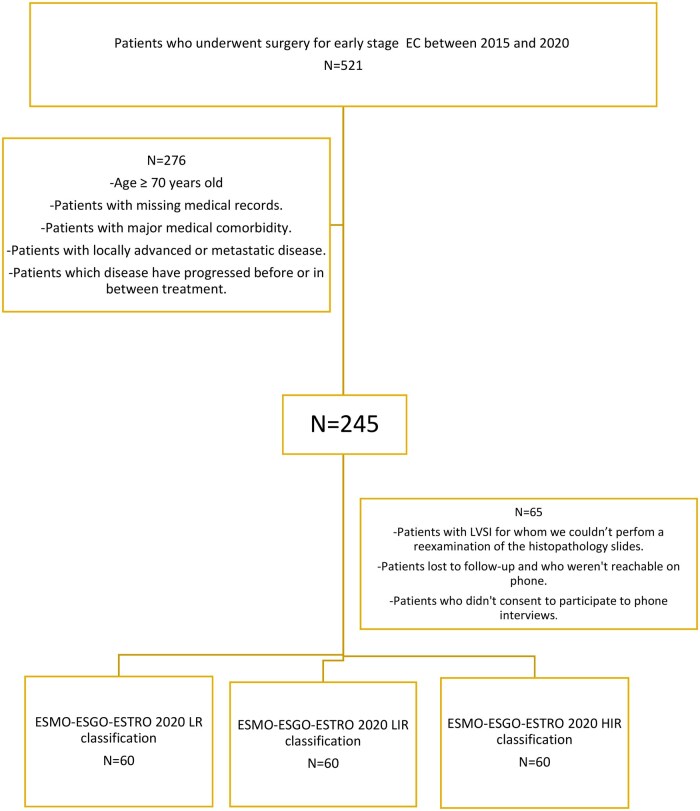
Patients’ selection in our research study.

This retrospective analytic study included women with endometrioid EC treated with primary surgery followed by adjuvant radiotherapy at SAI between 2015 and 2020. From 521 eligible cases, we selected 60 patients per risk group (low, low-intermediate, high-intermediate) to obtain comparable cohorts. Exclusion criteria were missing data, major comorbidities, inability to re-examine slides with lymphovascular space invasion (LVSI), progression before or during treatment, or refusal to participate. Non-endometrioid (type II) carcinomas were excluded (Figure 1).

### Data collection and ethics

Clinicopathologic and treatment data were abstracted from medical records and brachytherapy charts (Supplemental Dataset S1). Follow-up information and late toxicities were collected from outpatient visits or telephone interviews; late toxicities were graded using CTCAE v5 (Supplemental Dataset S2). All participants provided written consent for participation; verbal consent was obtained during telephone interviews.

### Histopathology and risk classification

Because resources were limited, histologic re-examination targeted only slides showing LVSI on the initial report. Two experienced gynecologic pathologists independently reviewed 11 slides to classify LVSI as focal (1–2 vessels) or extensive (> 2 vessels); discordances were resolved by consensus. Concordance between biopsy and hysterectomy grade is known to be modest (about 70%), but resources precluded systematic review of all 180 cases. Tumors were staged according to International Federation of Gynecology and obstetrics (FIGO) 2009, and patients were stratified into risk groups (low, low-intermediate, high-intermediate) using the ESGO–ESTRO–ESP 2020 criteria (Table S1). In this system, grade 1–2 endometrioid tumors are considered low-grade, whereas grade 3 tumors are high-grade. Grade 3 endometrioid carcinomas were allocated to the intermediate or high-intermediate risk groups depending on stage and LVSI.^1^

### Treatment and endpoints

Adjuvant therapy was delivered according to departmental practice. Radical pelvic lymphadenectomy was routinely performed because sentinel node mapping was not available. Adjuvant options included vaginal brachytherapy (VBT), external-beam radiotherapy (EBRT), or combined VBT + EBRT. We assessed whether the delivered treatment conformed to the 2021 ESGO–ESTRO recommendations. We defined 3 endpoints:

- **Conformal Treatment Group:** This group includes patients who received treatment that aligns with the established international treatment recommendations for their respective risk groups.
- **Over-Treatment Group:** Patients in this group received treatment that exceeded the recommendations specified for their risk group. They may have received more aggressive treatment than what was deemed necessary based on their risk factors.
- **Under-Treatment Group:** Patients in this group received treatment that was below the recommendations set for their specific risk group. This implies that their treatment might have been less intensive or comprehensive than suggested by the guidelines.

### Statistical analysis

Statistical analyses were performed using IBM SPSS v26. Survival curves were estimated with the Kaplan–Meier method and compared using log-rank tests. Multivariate analyses used Cox regression. A *P*-value < .05 was considered significant.

## Results

### A. Descriptive study

#### Epidemiological and clinical data

The distribution of endometrial cancer diagnoses in our study population across the years is as follows: 18 patients in 2015, 33 in 2016, 18 in 2017, 22 in 2018, 64 in 2019, and 25 in 2020.

The median age within the cohort was 60 years old, with an age spectrum from 35 to 69 years old. Endometrial carcinoma was diagnosed in 52.8% (*n* = 95) of cases among patients aged 60 to 69, while 47.2% (*n* = 85) of the patients were under the age of 60. Hypertension was observed in 50.6% (*n* = 91) of cases, diabetes in 31.7% (*n* = 57) of cases, and cardiopathy in 6.1% (*n* = 11) of cases. Median duration of hormonal exposure, determined as the period between menarche and menopause, was 28 years with interquartile range (IQR) [35-43], 43.1% have taken oral contraception, and nulliparity was observed in 24.5% of cases. A history of hormone-dependent cancer within the family was identified in 12.8% (*n* = 23) of cases, whereas 87.2% (*n* = 157) of patients reported no family history of cancer. Only 1.1% (*n* = 2) of patients had a smoking habit. In 16.7% (*n* = 30) of cases, the Body Mass Index (BMI) was equal to or exceeding 30, classifying them as obese. The rest of the population was evenly distributed between the healthy weight and overweight ranges, with 41.65% (*n* = 75) of patients falling into each of these categories.

#### Surgical data

All patients included underwent surgery first, resulting in 3 distinct approaches; total abdominal hysterectomy (TAH) along with bilateral salpingo-oophorectomy (BSO) in 45 patients, TAH and BSO, with a lymph node dissection (LND) in 104 patients, colpectomy, TAH, BSO, and LND in 31 patients. Our patients’ disease was classified according to the FIGO 2009 classification. FIGO IA comprised 38.4% (*n* = 69) of patients, FIGO IB included 36.1% (*n* = 65) of patients, and FIGO II included 25.5% (*n* = 46) of patients.

#### Histopathological data

The definitive histopathology results in our cohort showed a median tumor size of 35 mm, varying from 3 to 80 mm, grade 2 was observed in 51.1% (*n* = 92) of cases, while the remaining 48.9% (*n* = 88) were grade 1, myometrial invasion < 50% was observed in 54.5% (*n* = 97) of cases and it was absent in 2.8% (*n* = 5) of cases. No LVSI was found in 87.8% (*n* = 158) of cases, extensive LVSI was found in 8.3% (*n* = 15) of cases, and focal LVSI was noted in 3.9% (*n* = 7) of cases. P53 mutation data were available for only 2 patients, and in both cases, it was not mutated (Table 1).

**Table 1.** Tumor characteristics in our cohort.

Characteristics		HIR	LIR	LR	Total	*P*-value
**Endometrioid ADK**	*n* (%)	60 (100)	60 (100)	60 (100)	180 (100)	
**Grade I**	*n* (%)	29 (31.5)	32 (34.7)	31 (51.6)	92 (51.1)	9
**Grade II**	*n* (%)	31 (51.7)	28 (47.5)	29 (48.3)	88 (48.9)	
**Myo-**	*n* (%)	1 (1.7)	0	4 (6.8)	5 (2.8)	<.001
**Myo+** ** (<50%)**	*n* (%)	38 (63.3)	4 (6.8)	55 (93.2)	97 (54.5)	
**Myo+** ** (>50%)**	*n* (%)	21 (35)	55 (93.2)	0	76 (42.7)	
**Cervical invasion**	*n* (%)	45 (75)	0	0	45 (26)	<.001
**LVSI <0**	*n* (%)	45 (75)	53 (88.3)	60 (100)	158 (87.8)	<.001
**Focal LVSI**	*n* (%)	0	7 (11.7)	0	7 (3.9)	
**Extensive LVSI**	*n* (%)	15 (25)	0	0	15 (8.3)	
**LN dissection <0**	*n* (%)	45 (75)	52 (86.7)	35 (58.3)	135 (78.9)	<.001
**P53 < 0**	*n* (%)	1 (1.7)	1 (1.7)	0	2 (1.1)	.6

Abbreviations: ADK, adenocarcinoma; Extensive LVSI, >5 vessels; Focal LVSI, <5vessels; LN, lymph nodes; Myo, myometrial invasion.

#### Adjuvant treatment data

Vaginal brachytherapy alone was delivered in 50% (*n* = 90) of patients, 44.4% (*n* = 80) of patients received both VBT and pelvic EBRT, 5% (*n* = 9) of patients did not receive any adjuvant treatment, while 0.6% (*n* = 1) received pelvic EBRT alone. The majority of patients receiving pelvic EBRT, constituting 60.5% (*n* = 49), belonged to the HIR group. Followed by the LIR group accounting for 37% (*n* = 30) of cases, and the LR group comprised a smaller proportion at 2.5% (*n* = 2). Most patients were treated by conventional 2-Dimensional RT (75%), while the remaining 25% were treated using conformal 3-Dimensional RT. A normo-fractionated-RT of 45 Gy was delivered in 75.3% (*n* = 61), and 50 Gy was delivered in 24.7% (*n* = 20). At the radiation oncology department, a high dose rate (HDR)-VBT approach was implemented in the vast majority of cases, accounting for 94.8% (*n* = 161). In the remaining 5.2% of cases, Low Dose-Rate (LDR)-VBT brachytherapy was utilized due to either issues with the BT source or the patient’s residence in a remote location. Different VBT dosage regimens were administered. From the total population, 11.9% (*n* = 7) of individuals from the HIR group received chemotherapy. The chemotherapy was administered concurrently with pelvic EBRT in all 7 cases, and cisplatin was the chemotherapy agent used.

### B. Analytic study

#### Risk groups reclassification according to the ESMO–ESGO–ESTRO 2020 classification

**Figure 2.**
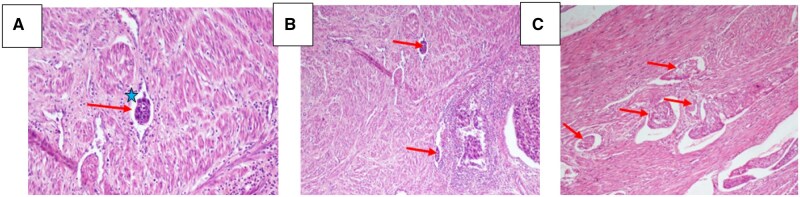
Histological slides taken from patients in our cohort showing in (A), 1 involved vessel (arrow) with carcinomatous cell clusters (asterisk) (HE×20), in (B) 2 involved vessels (HE×10), and in (C) 5 involved vessels (HE×20). HE, hematoxylin eosin

Eleven histopathological slides underwent re-examination to precisely assess the number of invaded vessels (Figure 2). Among these slides, 7 exhibited focal LVSI, leading to the reclassification of the disease into the LIR group, while 4 displayed extensive LVSI, reclassifying them into the HIR group.

#### Conformity to actual international treatment guidelines

It was observed that 47.2% (*n* = 85) of patients underwent treatment that aligned with the recommended guidelines, over-treatment was noted in 47.2% (*n* = 85) of cases. Suboptimal treatment was delivered for 5.6% (*n* = 10) of patients within the HIR group. Under-treatment was associated with patients’ non-compliance, obesity, grade 3 diarrhea following pelvic EBRT, and patient’s refusal of adjuvant VBT due to her virginity. The multi-disciplinary team’s decision to select adjuvant treatment for patients currently deemed over-treated was based on factors such as advanced age, the presence of myometrial invasion, and LVSI (Table 2).

**Table 2.** Conformity to actual international guidelines in our study population.

			HIR	LIR	LR	*N* (%)
**Status**	Under-treatment	*n* (%)	10 (16.7)	0	0	10 (5.6)
	Conformal	*n* (%)	50 (83.3)	29 (48.3)	6 (10)	85 (5.6)
	Over-treatment	*n* (%)	0	31 (51.7)	54 (90)	85 (47.2)
**Total**		*N* (%)	60 (100)	60 (100)	60 (100)	180 (100)

#### Follow-up and outcomes

Notably, 22.8% of cases were impacted by a limitation in data availability due to patients who were lost to follow-up. Median follow-up was 52.5 months, with extremes ranging from 25 and 102 months. The difference between different risk groups in terms of treatment outcomes was significative, with a *P*-value = .04. At a 3-year follow-up, our study showed that 71.1% (*n* = 128) of the population achieved disease control, characterized by the absence of both locoregional and metastatic disease. In 4.4% of cases (*n* = 8), there was locoregional relapse. In 1.7% of cases (*n* = 3), metastatic disease was observed. Out of the 11 patients who experienced disease relapse, either locoregionally or through metastatic disease, 4 were over-treated, 5 were conformally treated, and 2 were under-treated. Among the patients who experienced locoregional relapse or metastatic disease, 4 had EC classified in the LR group. All 4 patients underwent pelvic LND in addition to TAH + BSO, and out of them, 3 were categorized as over-treated, as they received adjuvant VBT. The 3 of them experienced vaginal relapse. The remaining patient was conformally treated, meaning she did not receive any adjuvant treatment and was closely observed. However, this patient developed pulmonary metastatic disease. Among the under-treated group, 6 showed disease control, 2 were lost to follow-up, and 2 exhibited vaginal relapse, which represents 25% of the under-treated group. Among the conformally treated patients, 60 showed disease control, 9 were lost to follow-up, and 4 patients exhibited either locoregional or metastatic relapse, which represents 5.3% of the conformally treated group.

Out of the over-treated patients, 62 showed disease control, 9 were lost to follow-up, and 5 patients showed either locoregional or metastatic relapse, which represents 6.6% of the over-treated group. In the study population, 4 patients experienced mortality events. Among these, 3 individuals were classified in the LIR group and were over-treated with adjuvant VBT + EBRT. One patient died from a cardiac stroke, while the other 2 faced mortality due to pulmonary metastatic disease after 10 and 36 months, respectively. The last patient, who belonged to the HIR group and received an undertreated regimen, developed grade 3 gastrointestinal toxicity that necessitated stopping EBRT; she died 2 years later.

#### Late toxicity

Treatment-related late toxicities were comprehensively assessed across the CTCAE V.5. grading system. For the Gastro-Intestinal (GI) system, it was observed in 26.7% (*n* = 48) of the total population. Constipation was observed in 17 patients (35.4%) after other causes were excluded. Abdominal or pelvic pain occurred in 13 cases (27.1%), diarrhea (after ruling out alternative causes) in 10 cases (20.8%), and abdominal distension in 8 patients (16.7%). Genito-Urinary (GU) toxicities were observed in 28.3% (*n* = 51) of the study population. Urinary frequency was noted in 35.3% (*n* = 18) cases, non-infective cystitis in 25.5% (*n* = 13) of cases, urinary incontinence in 21.6% (*n* = 11) of cases, and urinary retention in 17.6% (*n* = 9) of cases. Sexual-related toxicities were also documented in 12.8% (*n* = 23) among the 180 patients. Dryness in the vaginal area was encountered in 65.2% (*n* = 15), while 14.3% (*n* = 3) patients had inflammation, 14.3% (*n* = 3) experienced bleeding, and 9.5% (*n* = 2) reported pain in the vaginal region (Table 3).

**Table 3.** Summary of late toxicities in our cohort.

		Under-treatment	Conformal	Over-treatment	*P*-value
**Late toxicities**					
**Gastro-intestinal**	**Abdominal distension**				.088
	Grade 1	1 (13)	0	4 (50)	
	Grade 2	0	2 (25)	1 (13)	
	**Abdominal pain**				.033
	Grade 1	1 (8)	3 (23.1)	6 (46.2)	
	Grade 2	0	3 (23.1)	0	
	**Constipation**				.002
	Grade 1	1 (6)	1 (6)	7 (41.2)	
	Grade 2	1 (6)	5 (29.4)	2 (11.8)	
	**Diarrhea**				.042
	Grade 1	0	1 (10)	2 (20)	
	Grade 2	0	1 (10)	4 (40)	
	Grade 3	0	1 (10)	0	
**Genito-urinary**	**Non-infective cystitis**				.2
	Grade 1	1 (8)	3 (23.1)	4 (30.8)	
	Grade 2	0	2 (15.4)	3 (23.1)	
	**Urinary frequency**				.04
	Grade 1	1 (6)	2 (11.1)	5 (27.8)	
	Grade 2	0	4 (22.2)	6 (33.3)	
	**Urinary retention**				.03
	Grade 1	0	1 (11.)	0	
	Grade 2	0	0	8 (88.9)	
	**Urinary incontinence**				.2
	Grade 1	0	4 (36.4)	4 (36.4)	
	Grade 2	0	1 (9.1)	2 (18.2)	
**Sexual**	**Vaginal inflammation**				.048
	Grade 1	0	0	2 (66.7)	
	Grade 2	0	1 (33.3)	0	
	**Vaginal pain**				.15
	Grade 1	0	0	1 (50)	
	Grade 2	0	1 (50)	0	
	**Vaginal dryness**				.2
	Grade 1	1 (7)	5 (33.3)	3 (20)	
	Grade 2	0	2 (13.3)	4 (26.7)	
	**Vaginal bleeding**				.15
	Grade 1	0	1 (33.3)	1 (33.3)	
	Grade 2	0	0	1 (33.3)	

#### Survival outcomes

The 2-year Overall Survival (OS) rate was calculated at 92%, and the 3-year OS was at 88.6%, the 2-year Disease-Free Survival (DFS) rate was calculated at 83.5%, and the 3-year DFS was at 81.3%.

For under-treated patients, the 2-year OS rate was estimated at 80%, while the 3-year OS rate was at 60%. The 2-year OS for conformally treated patients was estimated at 87.1% and the 3-year OS rate was estimated at 87.1%. While the 2 and 3-year OS among over-treated patients were at 94.2% and 92.3%, respectively. The associated *P*-value was .098. Regarding the 2.3-year DFS, over-treated patients had a rate of 85.2% and 83.3%, respectively, while conformally treated patients’ rate was at 83.9%and 83.9%, respectively. For under-treated patients, the 2 and 3-year DFS rates were estimated at 50% each. The *P*-value for this comparison was.125, indicating that there was no statistically significant difference in terms of DFS between these groups.

#### Logistic regression analysis of survival factors

In this analysis, the variables examined exerted an impact on the 3-year DFS ranging from a minimum of 64.6% (as per Cox and Snell’s R^2^) to a maximum of 100% (according to ­Nagelkerke’s R^2^). Among these variables, tumor size emerged as statistically significant with a *P*-value of .003 and an odds ratio (OR) of 0.915; 95% CI, 0.875-0.966. This finding suggests an inverse proportionality between tumor size and 3-year DFS. However, none of the other factors were found to be statistically significant in their association with 3-year DFS.

After excluding women with a follow-up under 5 years, epidemiologic variables (age, endogenous and exogenous, hormonal exposure, parity, diabetes, BMI) exerted a notable influence on the 5-year DFS, elevating it from 59.4% to 82.1%. Among these variables, only age demonstrated statistical significance, with a *P*-value of .039.

Treatment variables demonstrated an impact on 5-year DFS ranging from 33.9% to 50.3%. Among these variables, 3 factors were found to be statistically significant:

The impact of adjuvant treatment on 5-year DFS was assessed by categorizing patients into different treatment groups, including those who received no adjuvant treatment, those treated with VBT alone, those who received pelvic EBRT alone, and those who received pelvic EBRT followed by VBT. The analysis revealed a statistically significant association (OR: 2.78; 95% CI, 2.56-2.99; *P* = .036). This implies that patients who underwent adjuvant treatment (whether by VBT alone, EBRT alone, or both EBRT and VBT) had a 2.78 times higher likelihood of experiencing improved 5-year DFS compared to those who did not receive adjuvant treatment. In other words, adjuvant treatment appears to play a crucial role in enhancing the likelihood of long-term DFS at the 5-year mark.

When choosing treatment-related late toxicity as dependent variable, variables such as age, adjuvant treatment, EBRT delay, EBRT dose, and over-treatment collectively contributed to the installation of toxicity by a range of 30% to 49.5%. However, it is noteworthy that none of these variables exhibited independent statistical significance.

#### Multivariate analysis of the survival

The results of the multivariate analysis using the Cox Regression technique demonstrated some highly significant findings. Specifically, variables such as myometrial invasion, treatment conformity, and adjuvant treatment were associated with very low *P*-values, all of which were less than.001. This indicates that these variables play a noteworthy role in influencing not only DFS but also OS outcomes.

#### Multivariate analysis of late toxicity

Among all late toxicities (GI, GU, sexual), both over and conformal treatment showed high significance with associated *P*-values <.001. Under-treatment did not exhibit any significant late toxicity in this study (Table 4).

**Table 4.** Multivariate analysis results in terms of late toxicity.

		*P*	OR	CI	
				Low	Upper
**Abdominal pain**	Over-treatment	<.001	6993	2976	16 393
	Conformal	<.001	5495	2304	13 158
**Constipation**	Over-treatment	<.001	4222	2042	8732
	Conformal	<.001	5500	2305	13 126
**Diarrhea**	Over-treatment	<.001	7000	2976	16 466
	Conformal	<.001	8750	3110	24 619
**Cystitis non infective**	Over-treatment	<.001	5857	2628	13 055
	Conformal	<.001	6800	2660	17 386
**Urinary frequency**	Over-treatment	<.001	3364	1716	6594
	Conformal	<.001	5500	2305	13 126
**Urinary incontinence**	Over-treatment	<.001	6833	2901	16 095
	Conformal	<.001	6800	2660	17 386
**Urinary retention**	Over-treatment	<.001	47 000	6485	340 649
	Conformal	<.001	38 000	5217	276 766
**Vaginal inflammation**	Over-treatment	<.001	23 000	5583	94 747
	Conformal	<.001	38 000	5217	276 766
**Vaginal pain**	Over-treatment	<.001	47 000	6485	340 649
	Conformal	<.001	38 000	5217	276 766
**Vaginal dryness**	Over-treatment	<.001	5857	2628	13 055
	Conformal	<.001	4571	2018	10 357
**Vaginal bleeding**	Over-treatment	<.001	23 000	5583	94 747
	Conformal	<.001	38 000	5217	276 766

## Discussion

### Epidemiological aspects of EC

The International Agency of Research on Cancer provided through the GLOBOCAN database of 2018, incidence and mortality of 36 cancers in 185 countries, across 20 world regions. In fact, Corpus uteri cancer showed an incidence of 2.1% across all cancer diagnoses, corresponding to an estimation of 382 069 new cases, while the mortality rate was 0.9% (*n* = 89 929). Additionally, it was described as the sixth most frequent cancer in females, and first gynecological cancer in developed countries, while developing countries report lower rates. Notably, it displayed a strong correlation with the socioeconomic development level of countries. In contrast, countries with low to medium Human Development Index (HDI) values had lower incidence rates.^2–4^ Data reveal a statistically significant surge in incidence among women aged 60 years and older at the time of diagnosis, with a *P*-value below.001 (*P* < .001). Duncan and colleagues’ research further supports this trend, indicating a consistent rise in incidence among women aged 60-64 years, increasing from 44.8 to 72.8 cases per 100 000 between 1985 and 2008.^5^ Literature and history suggest that endometrial cancer incidence has been associated with a variety of factors. These include both endogenous and exogenous hormonal exposures, obesity, hypertension, diabetes, early onset of menarche, nulliparity, late menopause, higher age (specifically, 55 years and older), and the use of tamoxifen.^5–11^

### Histopathology

Some histopathological parameters are required and reported after surgical resections for EC; tumor size, histotype, grade, depth of myometrial invasion, cervical stromal involvement, uterine serosal and adnexal involvement, parametrial and vaginal involvement, lymph node (LN) status with size of metastasis, biomarkers (Mismatch Repair (MMR), p53), and tumor stage (pathological Tumor, Node, and Metastasis classification (pTNM), FIGO).^9^

### Surgery

The established treatment for endometrial cancer involves a TAH with the BSO.^3^^,^^10^

For women seeking to preserve fertility, alternative approaches have been extensively reviewed, with minimally invasive techniques like laparoscopy or robot-assisted surgery being viable options, demonstrating safety in randomized clinical trials.^12–15^ These approaches are associated with shorter hospital stays and fewer postoperative complications compared to laparotomy. However, caution is advised against laparoscopic or robotic procedures in cases of bulky uterine malignant disease that may involve morcellation, as it can lead to tumor spillage, potentially increasing local or peritoneal recurrence and impacting survival.^3^

Surgical staging for endometrial cancer includes a thorough assessment of peritoneal surfaces, commonly involving omental and peritoneal biopsies in high-risk disease. The practice of assessing LNs during primary surgery varies widely, ranging from no nodal assessment to procedures such as sentinel node mapping or complete pelvic and aortic lymphadenectomy up to the renal vessels. Pelvic nodal dissection and pathological assessment remain crucial aspects of surgical staging, especially for apparent stage I EC, with criteria such as histology, grade, or Magnetic Resonance Imaging (MRI) findings influencing decisions. Para-aortic nodal assessment may be performed in specific high-risk cases, such as deeply invasive lesions, high-grade endometrioid EC, and non-endometrioid EC. However, despite historical practices, no survival advantage has been conclusively associated with staging lymphadenectomy in prospective, randomized clinical trials.^3^^,^^17^^,^^24^^,^^26–28^ The evolution of knowledge in gynecological oncology is gradually leading to surgical de-escalation in various areas, notably in the field of LN surgery. The use of sentinel LN in endometrial cancer, initially reserved for low and intermediate risks, is now expanded to all FIGO stages I and II,^18–20^ explaining why medical societies recommend discontinuing routine pelvic and para-aortic lymphadenectomy.^18^

### Risk grouping evolution

While the FIGO and Tumor, Node, and Metastasis (TNM) classifications are cornerstone frameworks for endometrial cancer staging, other pertinent prognostic factors, such as histological type and grade, patient age, tumor size, and LVSI, have been recognized.^21^^,^^22^ Consequently, risk stratification systems have emerged to amalgamate these additional prognostic factors, providing a more nuanced understanding of recurrence risk beyond the parameters defined by FIGO and TNM classifications. This categorization of endometrial cancer as per the risk groups has been an evolving subject. In 2020, a pivotal moment in gynecological oncology unfolded with the release of the fifth edition of the WHO Classification of Tumours: Female Genital Tumours.^23^ This edition boldly advocated for the integration of molecular parameters into the standard pathology reporting for EC. The winds of change also swept through the NCCN Clinical Practice Guidelines in Oncology, urging clinicians to delve deeper into ancillary studies testing for POLE mutations, MMR/Microsatellite Instability (MSI), and p53 status.^24^ This marked a departure from the traditional reliance solely on morphologic assessment of histologic tumor type. The collaborative efforts of the European Society of Gynaecological Oncology (ESGO), European Society for Radiotherapy and Oncology (ESTRO), and European Society of Pathology (ESP) further refined the landscape.^25^ Their joint guidelines not only integrated molecular subtypes into risk group assignments but also provided explicit recommendations for adjuvant therapy based on each risk group. This monumental shift led to a seismic impact on risk stratification, particularly with stage I–II POLEmut ECs now being deemed low risk, affording the option of forgoing additional therapy. Conversely, the guidelines identified p53abn ECs with any myometrial invasion as high risk, and intermediate risk if no invasion was present. The introduction of molecular classification, thus, not only altered the trajectory of EC management in 10% to 11% of all patients but also raised pertinent questions about the impact on survival, cost implications, and the potential for increased toxicity.^26^^,^^27^

### Risk-Based treatment strategies

#### LR patients

Patients with favorable risk profiles typically do not require adjuvant treatment. The risk of local recurrence in this group is generally less than 5%.^18^^,^^20^ A prospective randomized trial of 645 patients with LR EC treated with BT also showed no advantage for the use of adjuvant VB in terms of recurrence and OS, compared to no adjuvant treatment groups.^29^^,^^30^

#### LIR patients

For these patients, adjuvant BT is recommended to reduce the risk of recurrence.^18^^,^^20^ Large randomized trials^31^ with patients are considered IR, and a meta-analysis by Kong et al.^31^ found that EBRT reduced pelvic recurrence but had no benefit on OS.

#### HIR patients

Patients with HIR profiles may require a combination of treatments. Adjuvant VBT is recommended to mitigate the risk of vaginal recurrence, while adjuvant EBRT is advisable when LVSI is unequivocally positive, as it helps reduce the risk of pelvic recurrence.^18^^,^^20^ Some trials identified a subgroup of patients who benefited the most from adjuvant EBRT, named as the HIR group, where the risk of relapse was observed to be high enough to consider adjuvant RT.

#### Prognostic factors

Risk factors associated with an elevated incidence of LN recurrence and distant metastasis include positive LVSI or the presence of a grade 3 tumor.^32^ Cebecik Özmüş et al. found that DFS was most significantly influenced by stage and grade, while LVSI emerged as the most influential variable for OS.^33^ Jin et al. concluded that tumor grade was the most critical variable influencing both DFS and OS.^34^ In the PORTEC-1 study, lymphovascular space was not evaluated, and age was the most important risk factor, while the PORTEC-2 study, which included patients above 60 years, reported LVSI to be the most critical factor for local recurrences. In the study conducted by Cisek et al., age above 70 years emerged as the most significant variable influencing OS, while stage was identified as the most critical variable affecting DFS.^35^^,^^36^

## Conclusion

In Tunisia and other low and middle-income countries, limited access to molecular profiling constrains the potential for fully personalized care in EC management. Despite these barriers, there is a shared commitment to advancing treatment strategies, with a vision of incorporating molecular classification in the future to improve patient outcomes across resource-limited settings.

Future insights drawn from the biases of this study:

- Form comparable groups of under-treated, over-treated, and conformally treated patients.
- Standardize the use of VMAT.
- Global standardization of histological reports.
- Validate the predictive risk score with prospective studies.
- Incorporate molecular profiling.

## Supplementary Material

oyaf273_Supplementary_Data

## Data Availability

The date supporting the findings of this study are available within the article.

## References

[1] Peters EEM , León-Castillo A, Smit VTHBM, et al Defining substantial lymphovascular space invasion in endometrial cancer. Int J Gynecol Pathol. 2022;41:220-226. 34261899 10.1097/PGP.0000000000000806

[2] Gupta N , Pandey A, Dimri K, et al Endometrial cancer risk factors, treatment, and survival outcomes as per the European Society for Medical Oncology (ESMO)—European Society of Gynaecological Oncology (ESGO)—European Society for Radiotherapy and Oncology (ESTRO) risk groups and international Federation of Gynecology and Obstetrics (FIGO) staging: an experience from developing world. J Cancer Res Ther. 2023;19:701-707. 37470597 10.4103/jcrt.jcrt_1173_21

[3] Morice P , Leary A, Creutzberg C, Abu-Rustum N, Darai E. Endometrial cancer. Lancet. 2016;387:1094-1108. 26354523 10.1016/S0140-6736(15)00130-0

[4] Friberg E , Orsini N, Mantzoros CS, Wolk A. Diabetes mellitus and risk of endometrial cancer: a meta-analysis. Diabetologia. 2007;50:1365-1374. 17476474 10.1007/s00125-007-0681-5

[5] Duncan M , Seagroatt V, Goldacre M. Cancer of the body of the uterus: trends in mortality and incidence in England, 1985–2008. BJOG. 2012;119:333-339. 22082282 10.1111/j.1471-0528.2011.03201.x

[6] Terry P , Baron JA, Weiderpass E, Yuen J, Lichtenstein P, Nyrén O. Lifestyle and endometrial cancer risk: a cohort study from the Swedish Twin Registry. Int J Cancer. 1999;82:38-42. 10360818 10.1002/(sici)1097-0215(19990702)82:1<38::aid-ijc8>3.0.co;2-q

[7] Lucenteforte E , Bosetti C, Talamini R, et al Diabetes and endometrial cancer: effect modification by body weight, physical activity and hypertension. Br J Cancer. 2007;97:995-998. 17912243 10.1038/sj.bjc.6603933 PMC2360421

[8] Luo J , Beresford S, Chen C, et al Association between diabetes, diabetes treatment and risk of developing endometrial cancer. Br J Cancer. 2014;111:1432-1439. 25051408 10.1038/bjc.2014.407 PMC4183842

[9] Turashvili G , Hanley K. Practical updates and diagnostic challenges in endometrial carcinoma. Arch Pathol Lab Med 2024;147:78-98. 10.5858/arpa.2022-0280-RA 36943242

[10] Small W , Beriwal S, Demanes DJ, et al American brachytherapy society consensus guidelines for adjuvant vaginal cuff brachytherapy after hysterectomy. Brachytherapy. 2012;11:58-67. 22265439 10.1016/j.brachy.2011.08.005

[11] Janda M , Gebski V, Brand A, et al Quality of life after total laparoscopic hysterectomy versus total abdominal hysterectomy for stage I endometrial cancer (LACE): a randomised trial. Lancet Oncol. 2010;11:772-780. 20638899 10.1016/S1470-2045(10)70145-5

[12] Lu Q , Liu H, Liu C, et al Comparison of laparoscopy and laparotomy for management of endometrial carcinoma: a prospective randomized study with 11-year experience. J Cancer Res Clin Oncol. 2013;139:1853-1859. 24061340 10.1007/s00432-013-1504-3 PMC11824378

[13] Canlorbe G , Nikpayam M, Belghiti J. [Evolution of endometrial cancer surgery]. Rev Prat. 2022;72:738-741. 36511959

[14] Kilgore LC , Partridge EE, Alvarez RD, et al Adenocarcinoma of the endometrium: survival comparisons of patients with and without pelvic node sampling. Gynecol Oncol. 1995;56:29-33. 7821843 10.1006/gyno.1995.1005

[15] Todo Y , Kato H, Kaneuchi M, Watari H, Takeda M, Sakuragi N. Survival effect of para-aortic lymphadenectomy in endometrial cancer (SEPAL study): a retrospective cohort analysis. Lancet. 2010;375:1165-1172. 10.1016/S0140-6736(09)62002-X 20188410

[16] Benedetti Panici P , Basile S, Maneschi F, et al Systematic pelvic lymphadenectomy vs No lymphadenectomy in early-stage endometrial carcinoma: randomized clinical trial. J Natl Cancer Inst. 2008;100:1707-1716. 19033573 10.1093/jnci/djn397

[17] Rossi EC , Tanner E. Controversies in sentinel lymph node biopsy for gynecologic malignancies. J Minim Invasive Gynecol. 2021;28:409-417. 33359741 10.1016/j.jmig.2020.12.025

[18] Masson E. EM-Consulte. [cited 2023 Oct 2]. Désescalade chirurgicale en oncologie gynécologique. https://www.em-consulte.com/article/1473849/desescalade-chirurgicale-en-oncologie-gynecologiqu

[19] Parpex G , Lieng C, Koskas M. Less is more in endometrial cancer (SLN, conservative treatment, radical hysterectomy, molecular classification). Curr Opin Oncol. 2022;34:511-517. 35943439 10.1097/CCO.0000000000000874

[20] Murali R , Soslow RA, Weigelt B. Classification of endometrial carcinoma: more than two types. Lancet Oncol. 2014;15:e268-e278. 24872110 10.1016/S1470-2045(13)70591-6

[21] Bendifallah S , Canlorbe G, Raimond E, et al A clue gy improving the European Society of Medical Oncology risk group classification in apparent early stage endometrial cancer? Impact of lymphovascular space invasion. Br J Cancer. 2014;110:2640-2646. 24809776 10.1038/bjc.2014.237 PMC4037837

[22] Ren K , Wang W, Sun S, Hou X, Hu K, Zhang F. Recurrent patterns after postoperative radiotherapy for early stage endometrial cancer: a competing risk analysis model. Cancer Med. 2022;11:257-267. 34779587 10.1002/cam4.4423 PMC8704144

[23] Azaïs H , Lecointre L, Canlorbe G. Quelles nouveautés pour la prise en charge du cancer de l’endomètre? Le point sur les recommandations européennes de 2021. Gynécol Obstét Fertil Sénol. 2021;49:691-697. 33757927 10.1016/j.gofs.2021.03.005

[24] Höhn AK , Brambs CE, Hiller GGR, May D, Schmoeckel E, Horn LC. 2020 WHO classification of female genital tumors. Geburtshilfe Frauenheilkd. 2021;81:1145-1153. 34629493 10.1055/a-1545-4279 PMC8494521

[25] Abu-Rustum N , Yashar C, Arend R, et al Uterine neoplasms, version 1.2023, NCCN clinical practice guidelines in oncology. J Natl Compr Canc Netw. 2023;21:181-209. 36791750 10.6004/jnccn.2023.0006

[26] Concin N , Matias-Guiu X, Vergote I, et al ESGO/ESTRO/ESP guidelines for the management of patients with endometrial carcinoma. Radiother Oncol. 2021;154:327-353. 33712263 10.1016/j.radonc.2020.11.018

[27] Jamieson A , Huvila J, Thompson EF, et al Variation in practice in endometrial cancer and potential for improved care and equity through molecular classification. Gynecol Oncol. 2022 May;165:201-214. 35246332 10.1016/j.ygyno.2022.02.001

[28] Benichou J , Schwall C, Sastre-Garau X, et al Impact of the new molecular classification of endometrial cancer: a French cohort study. Gynecol Oncol. 2022;166:515-521. 35843738 10.1016/j.ygyno.2022.07.012

[29] Colombo N , Creutzberg C, Amant F, et al ESMO-ESGO-ESTRO consensus conference on endometrial cancer: diagnosis, treatment and follow-up. Ann Oncol. 2016;27:16-41. 26634381 10.1093/annonc/mdv484

[30] Sorbe B , Nordström B, Mäenpää J, et al Intravaginal brachytherapy in FIGO stage I low-risk endometrial cancer: a controlled randomized study. Int J Gynecol Cancer. 2009;19:873-878. 19574776 10.1111/IGC.0b013e3181a6c9df

[31] Fearon K , Strasser F, Anker SD, et al Definition and classification of cancer cachexia: an international consensus. Lancet Oncol. 2011;12:489-495. 21296615 10.1016/S1470-2045(10)70218-7

[32] Kong TW , Chang SJ, Paek J, Lee Y, Chun M, Ryu HS. Risk group criteria for tailoring adjuvant treatment in patients with endometrial cancer: a validation study of the Gynecologic Oncology Group Criteria. J Gynecol Oncol. 2015;26:32-39. 25376915 10.3802/jgo.2015.26.1.32 PMC4302283

[33] Bosse T , Peters EEM, Creutzberg CL, et al Substantial lymph-vascular space invasion (LVSI) is a significant risk factor for recurrence in endometrial cancer—a pooled analysis of PORTEC 1 and 2 trials. Eur J Cancer. 2015;51:1742-1750. 26049688 10.1016/j.ejca.2015.05.015

[34] Cebecik Özmüş D , Güzelöz Z, Şancı M. Evaluation of vaginal brachytherapy for treating early-stage endometrial cancer according to the European Society of Medical Oncology 2020 risk stratification. Turk J Obstet Gynecol. 2022;19:308-314. 36511631 10.4274/tjod.galenos.2022.47835 PMC9748865

[35] Jin M , Hou X, Sun X, Zhang Y, Hu K, Zhang F. Impact of different adjuvant radiotherapy modalities on women with early-stage intermediate- to high-risk endometrial cancer. Int J Gynecol Cancer. 2019;29:1264-1270. 31320487 10.1136/ijgc-2019-000317

[36] Cisek P , Kieszko D, Kordzińska-Cisek I, Kutarska E, Grzybowska-Szatkowska L. Retrospective analysis of intravaginal brachytherapy in adjuvant treatment of early endometrial cancer. Biomed Res Int. 2018;2018:7924153-7924159. 29682556 10.1155/2018/7924153 PMC5841031

