# Supplementary material for: Bridging adjuvant treatment gaps: low and intermediate risk endometrial carcinoma patients care in Salah Azaiez Institute in Tunisia
Source: Oncologist. 2025 Sep 17;30(9):oyaf273. doi: 10.1093/oncolo/oyaf273 (PMC12470651; doi:10.1093/oncolo/oyaf273)
Supplement: oyaf273_Supplementary_Data [file oyaf273_supplementary_data.docx]

**SUPPLEMENTAL MATERIALS**

**Supplemental Dataset S1: Patients’ Information Sheet**

Name

Phone number

Age

Age groups: < 60 years old and ≥ 60 years old

Past medical history:

-Hypertension

-Diabetes

-Cardiopathy

-Obesity

-Hormonal-dependent cancer in the family

Obstetrics and Gynecological past history

-Menarch

-Menopause

-Contraception

-Parity

Tabagism

First year of diagnosis

Chief complaint

MRI findings

Genital examination

Endometrial biopsy results

FIGO 2009 staging

Surgery

-Type of surgery

-Date of surgery

Definitive histopathology

-Tumor type

-Size

-Grade

-Myometrial invasion

-Cervical invasion

-LVSI

-Dissection

-P53

Previous risk group classification

Adjuvant treatment

-External Beam Radiotherapy (EBRT)

Delay of ERBT

EBRT course

Dose of RT

-VBT

Delay of VBT

Type of VBT

Dose of VBT

Fractions of VBT

-Chemotherapy

Disease control

OS

DFS

**Supplemental Dataset S2: Late toxicity Sheet**

-Gastro-intestinal:

Abdominal distension

CTCAE V.5. Grade

Abdominal pain

CTCAE V.5. Grade

Constipation

CTCAE V.5. Grade

Diarrhea

CTCAE V.5. Grade

-Genito-urinary:

Non-infective cystitis

CTCAE V.5. Grade

Urinary Frequency

CTCAE V.5. Grade

Urinary incontinence

CTCAE V.5. Grade

Urinary retention

CTCAE V.5. Grade

-Sexual:

Vaginal Inflammation

CTCAE V.5. Grade

Vaginal pain

CTCAE V.5. Grade

Vaginal dryness

CTCAE V.5. Grade

Vaginal bleeding

CTCAE V.5. Grade

Vaginal discharge

CTCAE V.5. Grade

**Supplemental Table S1: ESMO/ESGO/ESTRO 2020 risk classification**

**
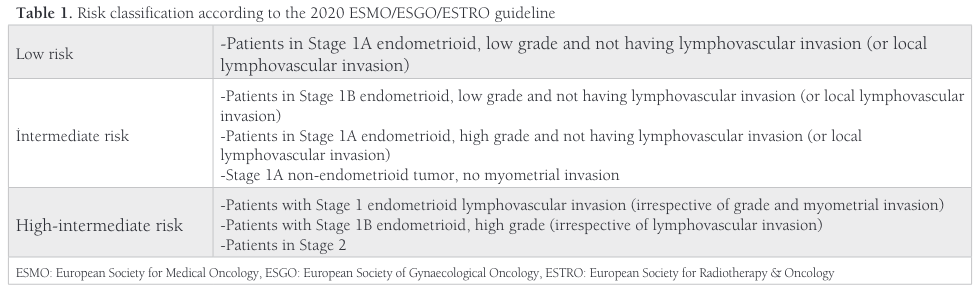
**
